# The anesthesia-to-incision interval accounts for most of the observed difference in decision-to-delivery time between urgency categories during emergency cesarean delivery: a single-center retrospective cohort study

**DOI:** 10.3389/fsurg.2026.1913793

**Published:** 2026-09-11

**Authors:** Guoqiang Zhao, Juan Dong, Yina Sun, Shengqiang Cai, Yuanyuan Zheng, Jinhong Chen, Hualin Xu

**Affiliations:** Department of Obstetrics, Shaoxing Maternity and Child Health Care Hospital, Shaoxing, China

**Keywords:** anesthesia, decision-to-delivery interval, emergency cesarean delivery, neonatal outcome, neuraxial anesthesia, urgency category

## Abstract

**Objective:**

Decision-to-delivery interval (DDI) is widely used to audit emergency cesarean delivery, but total DDI does not distinguish workflow performance from risk-based clinical decisions. This exploratory study compared DDI between urgency categories and quantified the contribution of individual process components to the observed difference.

**Methods:**

This single-center retrospective cohort study included 80 category I or II emergency cesarean deliveries between March 1, 2024, and October 31, 2025. DDI was divided into five mutually additive intervals. Between-category mean differences and component contributions were estimated using 50,000 stratified bootstrap resamples. Anesthesia type was considered a potential process-pathway variable because urgency may influence anesthetic strategy, which may in turn affect pre-incision workflow and total DDI.

**Results:**

Median DDI was 18.0 min, and 78 cases (97.5%) achieved delivery within 30 min. Median DDI was shorter in category I than category II cases [14.0 (10.0–16.0) vs. 19.0 (17.0–23.0) minutes; *P* < 0.001]. The reconstructed mean difference was 6.31 min (95% bootstrap CI, 3.85–8.73). The anesthesia-to-incision interval differed by 5.24 min (95% CI, 4.04–6.39) and accounted for 83.1% of the observed mean difference. General anesthesia was used in 73.7% of category I cases and 8.2% of category II cases. In the primary model adjusted for shift and major indication, category II urgency was associated with a 6.55-minute longer DDI (95% CI, 3.86–9.25).

**Conclusion:**

Most emergency cesarean deliveries met the 30-minute reference threshold, and the anesthesia-to-incision interval accounted for most of the observed DDI difference between urgency categories. These exploratory findings support risk-stratified, process-based DDI audit but do not establish a causal effect of any individual interval.

## Introduction

Emergency cesarean delivery requires coordinated obstetric, anesthesia, operating-room, and neonatal care when maternal or fetal condition necessitates urgent birth (1). Decision-to-delivery interval (DDI), defined as the time from the decision for cesarean delivery to fetal delivery, is commonly used to evaluate the timeliness of this response. The 30-minute threshold remains a familiar audit standard, although its relationship with neonatal outcomes is inconsistent across clinical settings (1–5).

Total DDI is an aggregate measure. It combines decision communication, team activation, transfer, anesthesia and pre-incision preparation, and operative delivery. Consequently, the same total DDI may arise from different workflows, and differences between patient groups may reflect both process performance and clinically appropriate risk-based decisions. Interpreting total DDI without accounting for urgency may therefore misclassify planned differences in care as operational delay (2, 4–7).

Urgency category is particularly relevant to anesthesia selection. Category I denotes an immediate threat to maternal or fetal life, whereas category II denotes urgent compromise without an immediate life-threatening condition (3, 6). General anesthesia is more often selected when the acceptable time window is shortest, while neuraxial anesthesia may be preferred when additional preparation time is compatible with maternal and neonatal safety (8, 9). Anesthesia type may therefore lie within the process pathway between urgency category and total DDI rather than act solely as a conventional confounder.

Process decomposition can locate an observed DDI difference within specific workflow intervals (10, 11). However, comparing separate *P* values for individual intervals does not quantify how much each interval contributes to the total difference. Estimating additive component differences with confidence intervals is necessary to distinguish the dominant observed component from smaller or opposing components.

We therefore compared total DDI and five prespecified process intervals between category I and category II emergency cesarean deliveries. We quantified each component's contribution to the observed mean DDI difference using bootstrap resampling and examined anesthesia selection, adjusted DDI associations, model diagnostics, shift timing, and early neonatal outcomes. The study was exploratory and hypothesis-generating, and no component was interpreted as an independently causal determinant of DDI.

## Materials and methods

### Study design and population

This single-center retrospective cohort study was conducted at Shaoxing Maternity and Child Health Care Hospital using records generated during routine clinical care. The study included emergency cesarean deliveries performed between March 1, 2024, and October 31, 2025. Clinical decisions, anesthesia selection, and treatment workflows were not altered for research purposes.

Potentially eligible category I and category II cases were identified from the electronic medical record system, operative anesthesia records, and related clinical databases. Of 95 records screened, 15 were excluded: 4 duplicates, 5 with missing key timestamps, 2 with non-calculable total or component intervals, and 4 that could not be reliably matched by patient identity and delivery time. The analytic cohort comprised 80 cases, including 49 from 2024 to 31 from 2025.

Complete-case analysis was required because the five mutually additive intervals could be calculated only when all six timestamps were available. No data were missing for variables used in the reported analyses among the 80 included cases, and no statistical imputation was performed.

### Eligibility criteria

Eligible cases were category I or category II emergency cesarean deliveries performed at the study hospital during the study period. Inclusion required complete documentation of the decision for surgery, telephone notification, operating-room entry, anesthesia start, skin incision, and fetal delivery, with a clinically plausible sequence.

Exclusion criteria were elective cesarean delivery converted to emergency surgery because of an intraoperative or perioperative event; missing or logically inconsistent timestamps that prevented calculation of DDI; confirmed intrauterine fetal death before admission; or unavailable complete records because of record sealing, external investigation, or another administrative restriction.

### Definitions and outcomes

The primary exposure was urgency category. The operating obstetric surgeon assigned the category according to the maternal and fetal condition at the time of the cesarean decision and documented it contemporaneously in the clinical record. Classification followed the institutional DDI management protocol, adapted from the Lucas classification. Category I indicated an immediate threat to maternal or fetal life requiring delivery as rapidly as possible. Category II indicated urgent maternal or fetal compromise without an immediate life-threatening condition.

Maternal and delivery variables included age, gestational age, parity categorized as multiparous or nulliparous, birth weight, shift timing, hypertensive disorder, comorbidity status, pre-existing neuraxial catheter, neuraxial contraindication, and BMI at delivery. Airway-risk assessment was not available in the retrospective records.

The primary outcome was total DDI, defined as the interval from the clinical decision for emergency cesarean delivery to fetal delivery. DDI was divided into five prespecified, mutually additive intervals: decision-to-notification, notification-to-operating-room entry, operating-room entry-to-anesthesia start, anesthesia-to-incision, and incision-to-delivery. Day shift was defined as 08:00–16:59 and night shift as 17:00–07:59 the following day, according to the decision time. Neonatal outcomes were 1- and 5-minute Apgar scores and neonatal intensive care unit (NICU) admission.

All six timestamps were entered manually and contemporaneously by the circulating nurse in the nursing information system. Timestamp resolution was one minute, and all entries referenced the hospital information-system clock. Source-record review found no evidence of systematic clock misalignment, although rounding and documentation-latency errors could not be excluded.

Anesthesia start was defined by technique: initiation of induction-drug administration for general anesthesia; completion of intrathecal local-anesthetic injection after successful puncture for *de novo* spinal or combined spinal-epidural anesthesia; and initiation of surgical-dose local-anesthetic administration through an existing labor epidural catheter. Neuraxial procedures were performed after operating-room entry. No conversion from neuraxial to general anesthesia was documented in the analytic cohort.

The recorded anesthesia-start event therefore differed by technique. Surgical skin preparation and draping did not overlap with the anesthesia procedure. Anesthesia readiness was determined clinically by the anesthesiologist, but a consistently structured anesthesia-ready timestamp was unavailable. The anesthesia-to-incision interval was consequently treated as a technique-specific composite pre-incision workflow measure rather than a standardized measure of anesthetic procedure duration or technical performance.

### Data collection and quality control

Trained investigators extracted data using a standardized form. Ambiguous entries were checked against original medical, nursing, anesthesia, operative, and delivery records. Records with clinically implausible timestamp sequences were excluded. The cleaned dataset retained the prespecified exposure, outcomes, process intervals, covariates, and neonatal variables.

### Statistical analysis

Continuous variables were assessed using the Shapiro–Wilk test and Q-Q plots. Normally distributed variables are reported as mean ± standard deviation and were compared using an independent-samples t test or Welch t test, as appropriate. Non-normally distributed variables are reported as median (interquartile range) and were compared using the Mann–Whitney U test. Categorical variables are reported as *n* (%) and were compared using the chi-square test or Fisher exact test. Tests were two-sided, with *P* < 0.05 considered statistically significant. Analyses were performed using IBM SPSS Statistics version 26.0 and R version 4.6.0.

Urgency category was the primary exposure. Anesthesia type was specified *a priori* as a potential process-pathway variable because urgency may influence anesthetic strategy, which may in turn affect pre-incision workflow and total DDI. Based on a prespecified conceptual framework, the primary multivariable model included urgency category, shift timing, and major cesarean indication but not anesthesia type, thereby estimating the total adjusted urgency-associated difference. Ordinary least squares was used because the target estimand was an adjusted arithmetic mean difference in minutes on the additive DDI scale. Given the small sample, covariates were prespecified and the models were deliberately parsimonious. A secondary exploratory model additionally conditioned on anesthesia type. HC3 robust standard errors, 95% confidence intervals, and *P* values were calculated. Formal causal mediation analysis was not performed because of the small sample and marked imbalance in anesthesia type across urgency categories.

For process decomposition, effects were arithmetic mean differences in minutes, calculated as category II minus category I. Each component's signed contribution was its mean difference divided by the total mean DDI difference. Uncertainty was estimated from 50,000 stratified nonparametric bootstrap resamples within urgency category, with patients resampled as intact rows. Percentile 95% confidence intervals were calculated for the total difference, component differences, and contributions. Direct bootstrap contrasts between the anesthesia-to-incision difference and each other component were adjusted using the Holm method.

Regression diagnostics included residual-versus-fitted and normal Q-Q plots; Shapiro–Wilk, Breusch-Pagan, White, and Ramsey RESET tests; externally studentized residuals; leverage; Cook's distance; variance inflation factors; and condition indices. Because DDI was positive and right-skewed, log-transformed ordinary least squares with HC3 standard errors and a gamma generalized linear model with a log link were fitted using the same covariates as the primary model. Log-scale coefficients were exponentiated and reported as DDI ratios.

For exploratory secondary analyses, effect estimates with 95% confidence intervals were reported alongside *P* values. Hodges–Lehmann location shifts were used for continuous outcomes, rank-biserial correlations for ordinal Apgar scores, and risk differences for binary outcomes. Confidence intervals were estimated using 50,000 nonparametric bootstrap resamples within the compared groups. Neonatal outcome analyses were descriptive and exploratory, and no multivariable neonatal model was fitted because the sample was small and baseline fetal risk was incompletely measured. No adjustment for multiple comparisons was applied outside the prespecified Holm-adjusted component contrasts; secondary and neonatal *P* values were interpreted descriptively.

### Ethics approval

The Ethics Committee of Shaoxing Maternity and Child Health Care Hospital approved the study and waived written informed consent because it used retrospective, de-identified clinical data.

## Results

### Study population and baseline characteristics

The analytic cohort included 80 emergency cesarean deliveries: 19 category I cases (23.8%) and 61 category II cases (76.3%). Maternal age, BMI at delivery, gestational age, parity, hypertensive disorder, comorbidity status, birth weight, and preterm birth are summarized in Table 1. Pre-existing functional labor epidural catheters were present in 2 of 19 category I cases (10.5%) and 11 of 61 category II cases (18.0%; Fisher exact *P* = 0.723).

**Table 1:** Baseline characteristics of category I and category II emergency cesarean deliveries.

Variable	Category I (*n* = 19)	Category II (*n* = 61)	Statistic	*P* value	95% CI
Maternal age, years	30.4 ± 5.3	30.5 ± 3.7	t = −0.099	0.921	[−2.249, 2.035]
Gestational age, weeks	38.2 (35.0–39.5)	38.3 (35.2–39.6)	Z = −0.023	0.982	
Multiparous, *n* (%)	9 (47.4)	22 (36.1)	chi-square=0.780	0.377	
Comorbidity present, *n* (%)	6 (31.6)	18 (29.5)	chi-square=0.064	0.801	
Birth weight, g	2,643.7 ± 776.1	2,690.8 ± 801.4	t = −0.225	0.820	[−463.3, 369.0]
Preterm birth, *n* (%)	6 (31.6)	16 (26.2)	chi-square=0.208	0.648	
BMI at delivery, kg/m2	27.4 ± 4.0	27.2 ± 3.2	t = 0.137	0.892	[−1.928, 2.204]
Hypertensive disorder, *n* (%)	1 (5.3)	9 (14.8)	Fisher exact test	0.437	
Pre-existing neuraxial catheter, *n* (%)	2 (10.5)	11 (18.0)	Fisher exact test	0.723	
Neuraxial contraindication, *n* (%)	0 (0.0)	0 (0.0)	Not estimable	NA	

Continuous variables are shown as mean ± standard deviation or median (interquartile range), according to distribution. Categorical variables are shown as *n* (%). No between-category test was performed for neuraxial contraindication because both groups had zero events.

**Figure 1:**
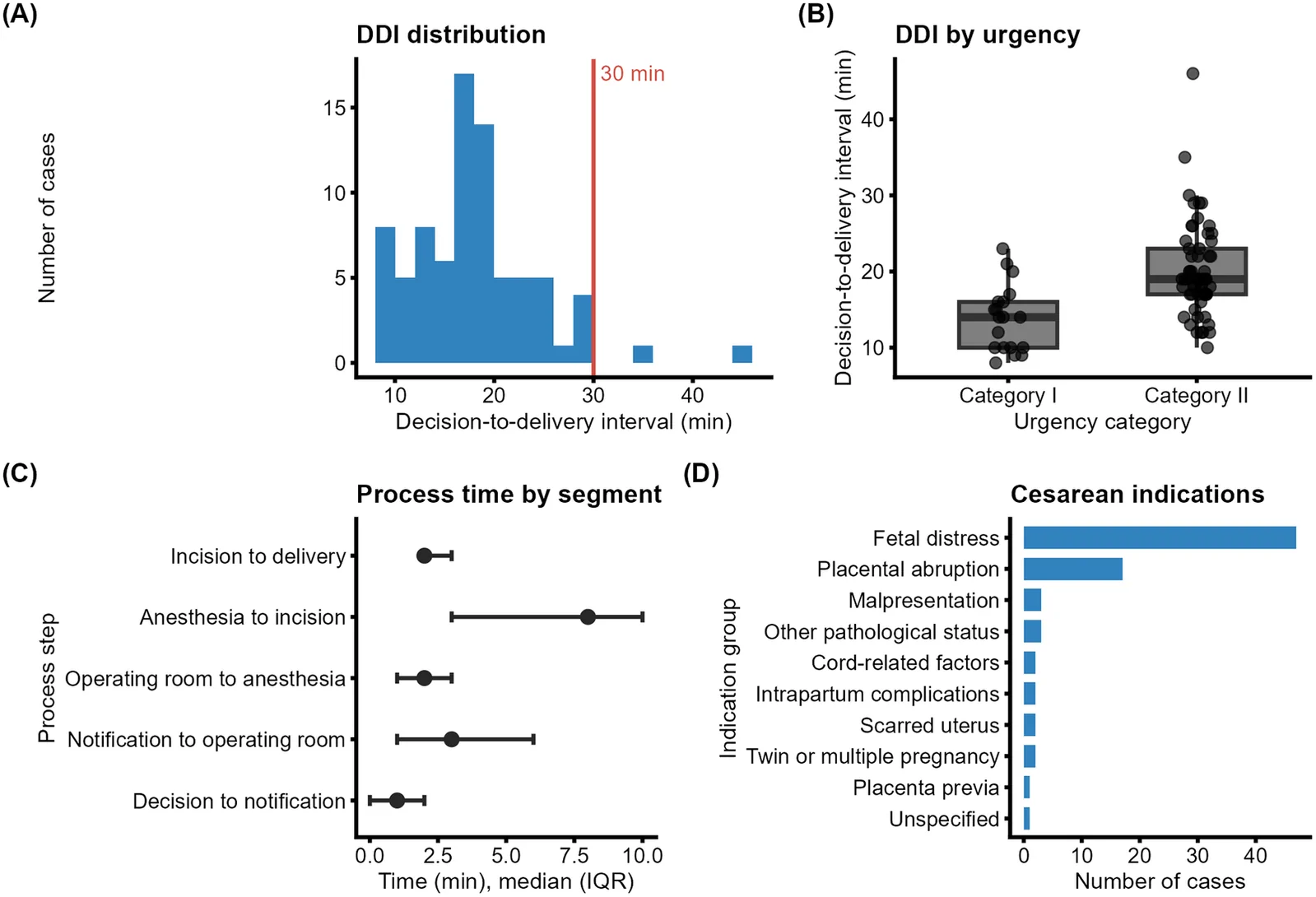
DDI performance and process structure in emergency cesarean delivery. **(A)** Distribution of DDI with the 30-minute reference line. **(B)** Comparison of DDI between category I and category II emergency cesarean deliveries. **(C)** Median and interquartile range of segmented DDI process intervals. **(D)** Distribution of main indications for emergency cesarean delivery.

In the overall cohort, median maternal age was 30.0 years (27.8–33.0), median gestational age was 38.2 weeks (35.2–39.5), and median birth weight was 2,835 g (2,315–3,300). Preterm birth occurred in 22 cases (27.5%), and 39 neonates (48.8%) were admitted to the NICU. The most common indications were fetal distress (47/80, 58.8%) and placental abruption (17/80, 21.2%) (Table 2; Figure 1D).

**Table 2:** Main indications for emergency cesarean delivery.

Indication	Overall (*n* = 80)	Category I (*n* = 19)	Category II (*n* = 61)
Fetal distress	47 (58.8)	12 (63.2)	35 (57.4)
Placental abruption	17 (21.2)	3 (15.8)	14 (23.0)
Malpresentation	3 (3.8)	0	3 (4.9)
Cord-related factors	2 (2.5)	2 (10.5)	0
Scarred uterus	2 (2.5)	0	2 (3.3)
Twin or multiple pregnancy	2 (2.5)	0	2 (3.3)
Other or unspecified	7 (8.8)	2 (10.5)	5 (8.2)

### Overall DDI and process profile

Median DDI was 18.0 min (14.0–22.0), mean DDI was 18.6 ± 6.3 min, and the range was 8.0–46.0 min. Seventy-eight cases (97.5%) had a DDI of 30 min or less, 58 (72.5%) had a DDI of 20 min or less, and 24 (30.0%) had a DDI of 15 min or less (Table 3; Figure 1A).

**Table 3:** Overall DDI and segmented process intervals.

Process indicator	Value
Decision-to-notification interval, min	1.0 (0.0–2.0)
Notification-to-operating-room entry interval, min	3.0 (1.0–6.0)
Operating-room entry-to-anesthesia start interval, min	2.0 (1.0–3.0)
Anesthesia-to-incision interval, min	8.0 (3.0–10.0)
Incision-to-delivery interval, min	2.0 (2.0–3.0)
Total DDI, median (interquartile range), min	18.0 (14.0–22.0)
Total DDI, mean ± standard deviation, min	18.6 ± 6.3
Total DDI, range, min	8.0–46.0
DDI <=30 min, *n* (%)	78 (97.5)
DDI <=20 min, *n* (%)	58 (72.5)
DDI <=15 min, *n* (%)	24 (30.0)

DDI was defined as the interval from decision for surgery to fetal delivery.

Median component durations were 1.0 min (0.0–2.0) for decision-to-notification, 3.0 min (1.0–6.0) for notification-to-operating-room entry, 2.0 min (1.0–3.0) for operating-room entry-to-anesthesia start, 8.0 min (3.0–10.0) for anesthesia-to-incision, and 2.0 min (2.0–3.0) for incision-to-delivery (Table 3; Figure 1C).

### Between-category DDI differences and process decomposition

Median DDI was 14.0 min (10.0–16.0) in category I and 19.0 min (17.0–23.0) in category II cases (*P* < 0.001; Figure 1B). The anesthesia-to-incision interval was also shorter in category I [3.0 (2.0–5.0) minutes] than category II cases [9.0 (7.0–11.0) minutes; *P* < 0.001]. The other four component intervals did not differ significantly between categories (Table 4).

**Table 4:** Process interval comparison between category I and category II emergency cesarean deliveries.

Time interval	Category I (*n* = 19)	Category II (*n* = 61)	Statistic	*P* value
Decision-to-notification	1 (0–1)	1 (0–1)	Z = −1.113	0.266
Notification-to-operating-room entry	2 (1–5)	3 (1–7)	Z = −1.282	0.200
Operating-room entry-to-anesthesia start	2 (1–7)	2 (1–3)	Z = −1.313	0.189
Anesthesia-to-incision	3 (2–5)	9 (7–11)	Z = −4.933	<0.001
Incision-to-delivery	2 (2–3)	2 (2–3.5)	Z = −0.925	0.355
Total DDI	14 (10–16)	19 (17–23)	Z = −4.105	<0.001

Non-normally distributed continuous variables are shown as median (interquartile range) and compared using the Mann–Whitney U test.

In the bootstrap decomposition, the reconstructed total mean DDI difference between category II and category I was 6.31 min (95% CI, 3.85–8.73). Component differences were 0.82 min (95% CI, −0.27–2.04; contribution, 13.1%) for decision-to-notification, 0.87 min (95% CI, −0.66–2.22; 13.9%) for notification-to-operating-room entry, −1.12 min (95% CI, −2.61–0.34; −17.8%) for operating-room entry-to-anesthesia start, 5.24 min (95% CI, 4.04–6.39; 83.1%) for anesthesia-to-incision, and 0.49 min (95% CI, −0.74–1.52; 7.8%) for incision-to-delivery (Table 5). The anesthesia-to-incision difference exceeded each other component difference after Holm correction (all adjusted *P* < 0.001).

**Table 5:** Bootstrap process decomposition of the between-category difference in decision-to-delivery interval.

Component	Category I mean, min	Category II mean, min	Mean difference (95% CI), min	Contribution, %
T1: decision-to-notification	0.95	1.77	0.82 (−0.27 to 2.04)	13.1
T2: notification-to-operating-room entry	3.16	4.03	0.87 (−0.66 to 2.22)	13.9
T3: operating-room entry-to-anesthesia start	3.63	2.51	−1.12 (−2.61 to 0.34)	−17.8
T4: anesthesia start-to-skin incision	3.32	8.56	5.24 (4.04 to 6.39)	83.1
T5: skin incision-to-delivery	2.79	3.28	0.49 (−0.74 to 1.52)	7.8
Total DDI	13.84	20.15	6.31 (3.85 to 8.73)	100.0

Between-category differences were calculated as category II minus category I. Confidence intervals were obtained from 50,000 stratified patient-level nonparametric bootstrap resamples. Component contributions were calculated on the arithmetic-mean scale as the signed component difference divided by the total mean DDI difference. Because contributions are signed, individual values may be negative or exceed 100% when differences in other components act in the opposite direction. For this decomposition, total DDI was reconstructed as the sum of the five confirmed component intervals after source-data reconciliation.

### Multivariable models and diagnostics

General anesthesia was used in 14 of 19 category I cases (73.7%) and 5 of 61 category II cases (8.2%), corresponding to an absolute risk difference of 65.5 percentage points (95% bootstrap CI, 43.1–84.9; *P* < 0.001) (Table 6). In the primary model adjusted for shift timing and major indication, category II urgency was associated with a 6.55-minute longer DDI than category I urgency (95% CI, 3.86–9.25; *P* < 0.001). Shift and major indication were not statistically significant (Table 7).

**Table 6:** Distribution of anesthesia type by urgency category.

Anesthesia type	Category I (*n* = 19)	Category II (*n* = 61)	Risk difference (95% CI), percentage points	*P* value
General anesthesia	14 (73.7)	5 (8.2)	65.5 (43.1 to 84.9)	<0.001
Neuraxial anesthesia	5 (26.3)	56 (91.8)		

Categorical variables are shown as *n* (%). The risk difference was calculated as category I minus category II for general-anesthesia use; its 95% CI was estimated using 50,000 bootstrap resamples.

**Table 7:** Primary and exploratory multivariable linear regression models for total DDI.

Variable	Primary total DDI beta (95% CI)	*P* value	Exploratory anesthesia-conditioned total DDI beta (95% CI)	*P* value
Category II vs. category I	6.55 (3.86 to 9.25)	<0.001	7.45 (4.40 to 10.50)	<0.001
General vs. neuraxial anesthesia	Not included		1.36 (−1.91 to 4.62)	0.415
Night vs. day shift	2.32 (−0.35 to 4.99)	0.088	2.28 (−0.37 to 4.94)	0.092
Fetal distress vs. other indications	−3.00 (−6.87 to 0.88)	0.128	−2.72 (−6.73 to 1.29)	0.184
Placental abruption vs. other indications	−1.50 (−5.40 to 2.40)	0.447	−1.46 (−5.38 to 2.47)	0.467

Beta coefficients are in minutes. The primary model included urgency category, shift, and major cesarean indication, but did not adjust for anesthesia type because anesthesia was conceptualized as a potential process-pathway variable. The secondary exploratory model additionally conditioned on anesthesia type. Reference groups were category I, day shift, other indications, and, in the exploratory model, neuraxial anesthesia. *P* values were based on HC3 robust standard errors. Primary total DDI model: R-squared=0.254, adjusted R-squared=0.215. Exploratory anesthesia-conditioned total DDI model: R-squared=0.259, adjusted R-squared=0.209. Positive-time sensitivity models used recorded total DDI and the same covariates as the primary model. The category II versus category I DDI ratio was 1.48 (95% CI, 1.26 to 1.74; *P* < 0.001) in log-transformed OLS and 1.48 (95% CI, 1.27 to 1.73; *P* < 0.001) in the gamma log-link model.

In the exploratory anesthesia-conditioned model, the category II coefficient was 7.45 min (95% CI, 4.40–10.50; *P* < 0.001). The coefficient for general versus neuraxial anesthesia was 1.36 min (95% CI, −1.91–4.62; *P* = 0.415) (Table 7).

For the primary model, residual Shapiro–Wilk *P* < 0.001, Breusch-Pagan *P* = 0.363, White test *P* = 0.819, and Ramsey RESET *P* = 0.780. Four observations exceeded the Cook's distance threshold of 4/*n* = 0.050; the maximum Cook's distance was 0.200, and maximum leverage was 0.133 (threshold, 0.125). After their exclusion, the urgency coefficient was 5.95 min (95% CI, 3.74–8.16; *P* < 0.001). Maximum VIF was 1.09 and maximum condition index was 1.38. For the anesthesia-conditioned model, residual Shapiro–Wilk *P* < 0.001, Breusch-Pagan *P* = 0.497, White test *P* = 0.977, and Ramsey RESET *P* = 0.624. The corresponding influence analysis yielded an urgency coefficient of 6.91 min (95% CI, 4.10–9.73; *P* < 0.001), maximum VIF of 1.92, and maximum condition index of 2.44 (Figure 3).

Sensitivity analyses using the recorded total DDI supported the primary association. In the log-transformed ordinary least-squares model, category II urgency was associated with a log-scale coefficient of 0.390 (95% CI, 0.227–0.552; *P* < 0.001), corresponding to a DDI ratio of 1.48 (95% CI, 1.26–1.74) relative to category I. In the gamma model with a log link, the corresponding coefficient was 0.395 (95% CI, 0.241–0.548; *P* < 0.001), corresponding to a DDI ratio of 1.48 (95% CI, 1.27–1.73).

### Exploratory analyses of shift timing and neonatal outcomes

Within category I, median DDI was 12.0 min (9.2–14.0) during day shifts and 15.0 min (10.0–17.0) during night shifts; the night-minus-day Hodges–Lehmann shift was 2.0 min (95% bootstrap CI, 0.0–7.0; *P* = 0.102). Within category II, the corresponding medians were 18.0 min (16.5–20.0) and 19.0 min (17.0–24.8), with a shift of 2.0 min (95% CI, −1.0–5.0; *P* = 0.145). Median DDI was 16.0 min (14.0–19.0) with general anesthesia and 19.0 min (16.0–22.0) with neuraxial anesthesia; the unadjusted neuraxial-minus-general shift was 2.0 min (95% CI, 0.0–5.0; *P* = 0.083) (Table 8).

**Table 8:** DDI analysis by shift and anesthesia type.

Analysis	Group	n	DDI, median (interquartile range)	HL shift (95% CI), min	*P* value
Shift within category I	Day shift	6	12.0 (9.2–14.0)	2.0 (0.0 to 7.0)	0.102
Shift within category I	Night shift	13	15.0 (10.0–17.0)		
Shift within category II	Day shift	31	18.0 (16.5–20.0)	2.0 (−1.0 to 5.0)	0.145
Shift within category II	Night shift	30	19.0 (17.0–24.8)		
Anesthesia type	General anesthesia	19	16.0 (14.0–19.0)	2.0 (0.0 to 5.0)	0.083
Anesthesia type	Neuraxial anesthesia	61	19.0 (16.0–22.0)		

Shift analyses were conducted within urgency category, and the anesthesia-type analysis was conducted in the overall cohort. Hodges–Lehmann (HL) shifts were defined as night minus day for shift analyses and neuraxial minus general for the unadjusted anesthesia analysis.

Category I cases had lower 1-minute Apgar scores than category II cases [8.0 (6.0–8.5) vs. 9.0 (8.0–9.0); rank-biserial correlation, −0.348; 95% bootstrap CI, −0.598 to −0.076; *P* = 0.014]. Median 5-minute scores were 9.0 in both categories, with a rank-biserial correlation of −0.267 (95% CI, −0.503 to −0.032; *P* = 0.038). NICU admission occurred in 13 of 19 category I cases (68.4%) and 26 of 61 category II cases (42.6%), corresponding to a risk difference of 25.8 percentage points (95% CI, 0.5–49.4; *P* = 0.049) (Table 9).

**Table 9:** Neonatal outcomes by urgency category.

Outcome	Category I (*n* = 19)	Category II (*n* = 61)	Effect estimate (95% CI)	*P* value
1-minute Apgar score	8.0 (6.0–8.5)	9.0 (8.0–9.0)	r_rb = −0.348 (−0.598 to −0.076)	0.014
5-minute Apgar score	9.0 (8.0–9.0)	9.0 (9.0–9.0)	r_rb = −0.267 (−0.503 to −0.032)	0.038
NICU admission, *n* (%)	13 (68.4)	26 (42.6)	RD = 25.8 pp (0.5 to 49.4)	0.049

Rank-biserial correlations (r_rb) were calculated as category I minus category II; negative values indicate lower scores in category I. The NICU risk difference (RD) was category I minus category II. Confidence intervals were estimated using 50,000 bootstrap resamples.

**Figure 2:**
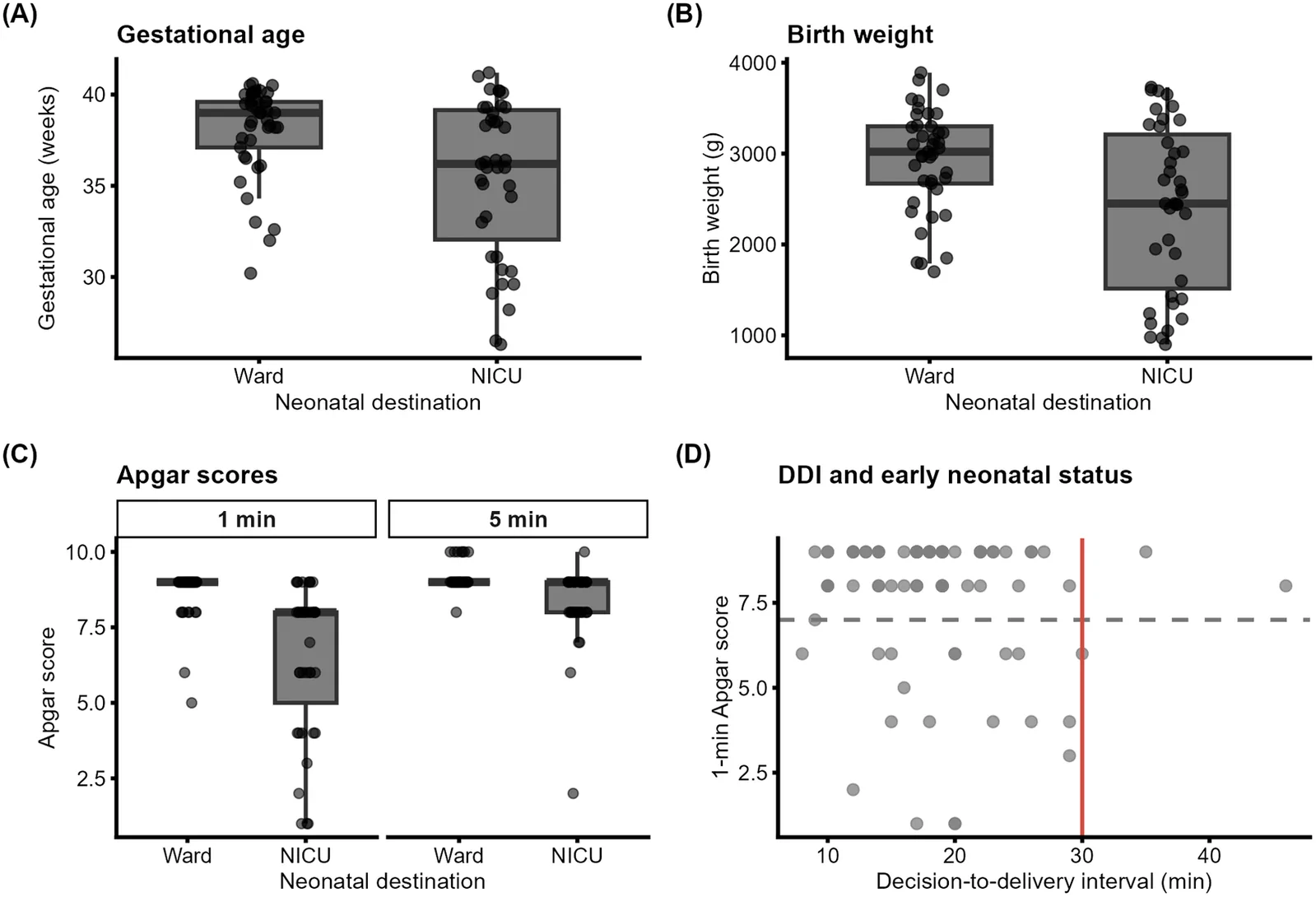
DDI and early neonatal outcomes. **(A)** Gestational age distribution by neonatal destination. **(B)** Birth weight distribution. **(C)** Distribution of 1-minute and 5-minute Apgar scores. **(D)** Scatter plot of DDI and 1-minute Apgar score.

Compared with neonates transferred to the ward, NICU-admitted neonates had a lower gestational age [36.2 (32.0–39.1) vs. 39.0 (37.1–39.6) weeks; Hodges–Lehmann shift, −1.9 weeks; 95% bootstrap CI, −3.8 to −0.4; *P* = 0.007] and lower birth weight [2,450 g (1,515–3,210) vs. 3,020 g (2,670–3,300); shift, −460 g; 95% CI, −840 to −100; *P* = 0.015]. Rank-biserial correlations for 1- and 5-minute Apgar scores were −0.645 (95% CI, −0.805 to −0.466; *P* < 0.001) and −0.528 (95% CI, −0.687 to −0.358; *P* < 0.001), respectively (Table 10; Figures 2A–C).

**Table 10:** Perinatal indicators stratified by neonatal postoperative destination.

Indicator	NICU (*n* = 39)	Ward (*n* = 41)	*P* value	Effect estimate (95% CI)
Gestational age, weeks	36.2 (32.0–39.1)	39.0 (37.1–39.6)	0.007	HL = −1.9 weeks (−3.8 to −0.4)
Birth weight, g	2,450.0 (1,515.0–3,210.0)	3,020.0 (2,670.0–3,300.0)	0.015	HL = −460 g (−840 to −100)
Total DDI, min	18.0 (14.5–23.5)	17.0 (14.0–20.0)	0.441	HL = 1.0 min (−2.0 to 3.0)
1-minute Apgar score	8.0 (5.0–8.0)	9.0 (9.0–9.0)	<0.001	r_rb = −0.645 (−0.805 to −0.466)
5-minute Apgar score	9.0 (8.0–9.0)	9.0 (9.0–9.0)	<0.001	r_rb = −0.528 (−0.687 to −0.358)

Groups were compared using the Wilcoxon rank-sum test. Hodges–Lehmann (HL) shifts and rank-biserial correlations (r_rb) were calculated as NICU minus ward, with 95% CIs estimated using 50,000 bootstrap resamples. This stratification was descriptive and does not imply causal inference.

**Figure 3:**
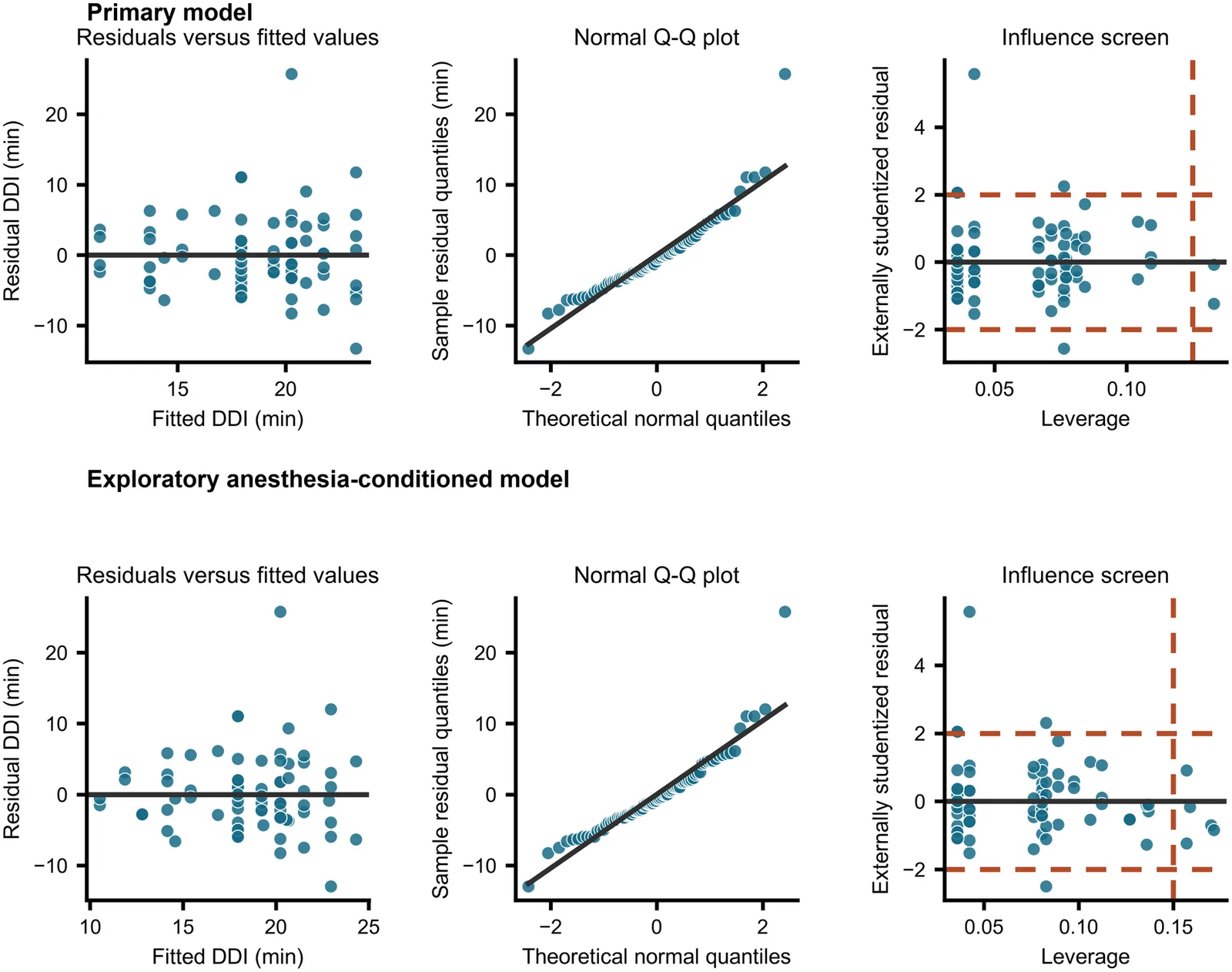
Regression diagnostic plots for the DDI regression models. **(A)** Primary multivariable model including urgency category, shift timing, and major cesarean indication. **(B)** Exploratory anesthesia-conditioned model additionally including anesthesia type. For each model, panels show residuals versus fitted values, normal Q-Q plots, and influence screening using leverage and externally studentized residuals.

Median DDI was 18.0 min (14.5–23.5) among NICU-admitted neonates and 17.0 min (14.0–20.0) among those transferred to the ward; the NICU-minus-ward Hodges–Lehmann shift was 1.0 min (95% bootstrap CI, −2.0–3.0; *P* = 0.441). Figure 2D shows the distribution of DDI and 1-minute Apgar scores.

## Discussion

### Principal findings

This exploratory cohort yielded three principal findings. First, 97.5% of emergency cesarean deliveries met the 30-minute reference threshold, and category I cases had shorter DDI than category II cases. Second, the anesthesia-to-incision interval accounted for 83.1% of the observed mean between-category difference. Third, anesthesia selection differed markedly by urgency category, while neonatal differences accompanied differences in gestational age, birth weight, and early clinical condition.

### Interpretation of the principal finding

The component analysis adds information that total DDI alone cannot provide. Previous studies have linked DDI to communication, operating-room availability, transfer, team coordination, anesthesia, urgency, and health-system resources (7, 10–16). In this cohort, the between-category differences in decision activation, transfer, operating-room entry to anesthesia start, and operative delivery were small and imprecisely estimated. By contrast, the anesthesia-to-incision difference was larger than every other component difference in direct bootstrap contrasts.

This concentration should not be interpreted as proof that the anesthesia-to-incision interval independently caused the total DDI difference. The interval began at different technique-specific events and ended at skin incision because a structured anesthesia-ready timestamp was unavailable. It therefore combined anesthetic preparation, readiness assessment, and subsequent pre-incision workflow. Its magnitude identifies where the observed time difference was located, but the available timestamps cannot separate the individual activities within that interval.

Anesthesia selection also formed part of the clinical pathway. Category I urgency was strongly associated with general anesthesia, whereas category II cases predominantly received neuraxial anesthesia. Urgency may influence anesthetic strategy, and technique-specific preparation may then influence pre-incision workflow (1, 8, 9). For this reason, the primary model did not condition on anesthesia type. The anesthesia-conditioned model was retained as an exploratory analysis, not as an estimate of an independent anesthesia effect or formal mediation. Its urgency coefficient was not attenuated, which further limits any simple mediation interpretation. Unmeasured fetal status, anesthesia indication, airway considerations, and clinician judgment may have influenced both strategy and timing (8, 9, 17).

### Clinical and quality-improvement implications

These findings favor risk-stratified, component-based DDI audit over a single undifferentiated threshold. Category I systems must preserve rapid activation and delivery capacity, whereas category II care must balance timeliness with anesthesia and perioperative safety. Within this framework, the anesthesia-to-incision interval can serve as a target for more detailed local process review, including anesthesia handoff, readiness documentation, and pre-incision coordination. It should not be used alone to rank anesthetic techniques or individual clinical performance.

### Interpretation of exploratory analyses

The shift analyses remained inconclusive. The estimated night-minus-day difference was 2 min in both urgency categories, but the confidence intervals included no difference and allowed clinically relevant prolongation, particularly in the six category I daytime cases. These estimates therefore do not establish equivalent day- and night-shift performance. Larger multicenter studies are needed to examine staffing, operating-room access, and team configuration across shifts (2, 18, 19).

Category I cases had lower Apgar score distributions and a 25.8-percentage-point higher observed NICU admission rate, although the confidence interval for the risk difference was wide. NICU-admitted neonates also had lower gestational age, lower birth weight, and lower Apgar score distributions, whereas the DDI location shift was small and imprecise. These descriptive findings are consistent with neonatal dispossition being strongly related to baseline maturity, fetal condition, and surgical indication (2, 5, 18, 20–23). Because baseline fetal risk was incompletely measured, no multivariable neonatal model was fitted, and multiple comparisons were not generally adjusted, the analyses do not establish the absence of a DDI effect or an independent effect of urgency or anesthesia.

### Strengths and limitations

A strength of this study was the additive decomposition of DDI using six contemporaneously recorded timestamps. The bootstrap analysis reported component effect sizes, confidence intervals, signed contributions, and direct contrasts. Secondary analyses also paired *P* values with effect estimates and confidence intervals, and positive-time sensitivity models quantified the stability of the primary urgency association. The analytic framework distinguished the total urgency-associated difference from the exploratory model conditioned on anesthesia type and included residual, influence, heteroskedasticity, and collinearity diagnostics.

The study also has important limitations. Its retrospective, single-center design and small sample, particularly the 19 category I cases, limited power, generalizability, covariate adjustment, and the stability of subgroup and influence analyses. Complete timestamp requirements may have introduced selection bias. Pre-existing neuraxial catheters were uncommon, and no neuraxial contraindications were recorded, precluding meaningful estimation of their independent associations. Residual confounding remains likely because detailed fetal status, anesthesia indication, airway risk, disease severity, and clinician judgment were not fully captured.

Timestamp entries were manual, had one-minute resolution, and may contain rounding or documentation-latency error. Technique-specific anesthesia-start definitions and the absence of a structured anesthesia-ready timestamp made the anesthesia-to-incision interval a composite measure. Retrospective records may also incompletely capture workflow overlap or unsuccessful anesthesia attempts. Finally, the neonatal and other secondary analyses were exploratory, multiple comparisons were not generally adjusted, and nonsignificant findings should not be interpreted as equivalence or absence of effect.

## Conclusion

In this single-center cohort, category I emergency cesarean deliveries had shorter DDI than category II deliveries, and 97.5% of all cases met the 30-minute reference threshold. The anesthesia-to-incision interval accounted for 83.1% of the observed mean between-category difference, identifying the pre-incision phase as the principal location of that difference without establishing causation. These exploratory findings support risk-stratified, process-based DDI audit and require confirmation in larger multicenter studies with more detailed anesthesia-readiness and fetal-risk data.

## Data Availability

The raw data supporting the conclusions of this article will be made available by the authors, without undue reservation.
